# Fatigue Behaviour of Brazed Joints for Heat Exchangers

**DOI:** 10.3390/ma17020479

**Published:** 2024-01-19

**Authors:** Blaž Hanželič, Jernej Kralj, Tonica Bončina, Branko Nečemer, Janez Kramberger, Roman Satošek, Srečko Glodež

**Affiliations:** 1 Faculty of Mechanical Engineering, University of Maribor, Smetanova 17, 2000 Maribor, Slovenia; blaz.hanzelic1@um.si (B.H.); jernej.kralj@student.um.si (J.K.); tonica.boncina@um.si (T.B.); branko.necemer@um.si (B.N.); janez.kramberger@um.si (J.K.); 2 Danfoss Trata d.o.o., Korenova 5, 1241 Kamnik, Slovenia; roman.satosek@danfoss.com

**Keywords:** brazed joint, fatigue, experimental testing, computational analyses, heat exchanger

## Abstract

The plate heat exchanger (PHE) is a component that provides heat to be transferred from hot water to domestic cold water without mixing them with high efficiency. Over the lifetime of the PHE, cyclic pressures act on the brazing points and the plates, and this may lead to fatigue failure. The fatigue behaviour of the PHE, designed using copper-brazed 316L stainless steel, was investigated in this study. First, the fatigue tests under the load ratio *R* = 0.1 were performed on the Vibrophore 100 testing machine to obtain the S-N curve of the analysed brazed joint. Based on the obtained experimental results, an appropriate material model of the analysed brazed joint has been created, which was validated with numerical calculation in the framework of a program code Ansys. A validated material model was then used for the subsequent numerical analysis of PHE. In order to carry out a numerical calculation using the finite element method (FEM), a three-dimensional model of the heat exchanger was created based on the previous scanning of PHE-geometry. Thereafter, the geometry was parameterised, which allowed us to perform parametric simulations (monitoring different responses depending on the input geometry). Numerical simulations were carried out in the framework of the Ansys 2023-R1 software, whereby the obtained results were analysed, and the responses were appropriately characterised according to previously determined load cases.

## 1. Introduction

In different engineering applications, different devices may be found to transfer heat from hot to cold water. One of them is a heat exchanger, which can be used for central heating of houses, heating of domestic water and many other purposes, including the provision for different kinds of industries, e.g., the food industry, the pharmaceutical industry, and others [1,2,3,4,5].

Due to high applicability, there are many different types of heat exchangers. Even though they all transfer heat, they use different methods to reach this goal. Heat exchangers may generally be divided into two main groups: recuperators and regenerators [6,7,8]. In the first case, the heat is transferred from hot to cold water simultaneously, while in the second case, a time delay due to the heat transferring method is used [9,10]. According to the aggregate state of the heat transferring medium, we also know about single-phase and multi-phase heat exchangers [11,12,13]. In this study, a single-phase heat recuperator (plate heat exchanger) will be analysed, considering the assumption that water will be used for both mediums. Furthermore, mediums do not mix with each other.

A plate heat exchanger (PHE) is a component which enables heat to be transferred from hot to cold medium without mixing them [14,15,16,17]. Here, the total heat transfer surface of channel plates is very large, which enables a high efficiency of such a component. However, there are many parameters (i.e., the thickness of the channel plate, water flow rate, number of channel plates, the thermal conductivity of plate material, etc.) which may influence the final functionality and real efficiency of PHE [18,19,20].

**Figure 1:**
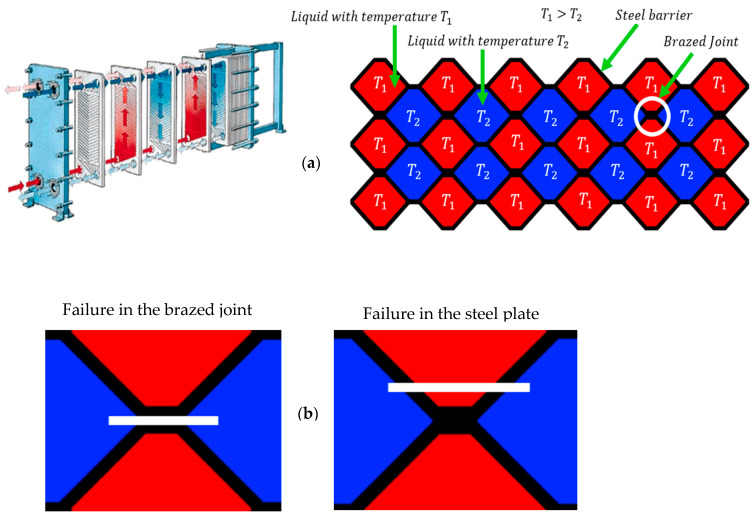
Schematic presentation of the brazed plate heat exchanger (**a**) and different types of failure (**b**).

PHEs are generally produced by vacuum brazing to join the channel plates to each other by using a filler material, which is usually copper, due to its ease of diffusion to steel, good wetting ability and corrosion resistance. This type of PHE consists of many parallel plates separating the hot and cold channels of PHE (see Figure 1a). The number of plates, plate thickness and overall size depend mainly on the power which is needed for a certain scope of application. The commonly used materials for PHE production are 316L and 304L stainless steels [21,22,23,24].

As mentioned above, vacuum brazing technology is often used in the PHE industry. Vacuum brazing is a jointing process that is accomplished at high temperatures, usually between 930 °C and 1230 °C, by using pure copper or nickel-based alloy as a filler material [25,26]. In this production technology, the naturally formed oxide layers on the base metals can be decomposed in a vacuum atmosphere at high temperatures, which provides high strength and less porosity of brazed joints. In order to achieve a high-quality brazed joint, the braising parts must be closely fitted, and the base metal surfaces must be well-cleaned. Here, the suitable clearance between braising parts should not exceed 0.1 mm in order to reach a good capillary effect. Braising surface cleanliness is very important since contaminations on the surface can lead to insufficient wetting [27,28,29].

The brazed joint itself represents a possible weak spot where failure may occur due to external loading (i.e., fluid pressure) acting on the brazed channel plates. However, the failure may occur directly in the brazed joint or in the base steel plate (see Figure 1b). In the first case (also considered in this study), failure occurs as a consequence of pressure inside the liquid medium, which results in the dynamic tensile forces acting on the brazed joint. At the moment when the failure occurs in one of the PHE’s brazed joints, the tensile stress on the remaining brazed joints in the surrounding area increases, which leads to a higher possibility of failure in those joints, and, consequently, reduces fatigue life of PHE [30].

In addition to the mechanical properties of the base material (stainless steel) and braze material (copper), the strength of brazed joints is also influenced by the dynamic character of external load (i.e., loading ratio *R* = σ_min_/σ_max_), which varies with different applications of PHEs [31]. Furthermore, there are also some other influencing magnitudes which can have a significant impact on the fatigue behaviour of PHE: surface roughness, loading frequency, notch effects, temperature, etc. [32].

This study focused on the computational analyses of brazed heat exchangers loaded with dynamic internal pressure. The computational model is based on the previously developed material model of the analysed brazed joint. In order to carry out a numerical calculation in the framework of Ansys software [33], a three-dimensional model of the heat exchanger was created based on the previous scanning of PHE geometry. Thereafter, the geometry was parameterised, which allowed us to perform parametric simulations, i.e., monitoring different responses depending on the input geometry. The obtained computational results are critically evaluated according to previously determined load cases. From that respect, the main contribution of this research is the developed material model for the subsequent computational analyses of plate heat exchangers.

## 2. Materials and Methods

### 2.1. Material and Specimen Geometry of the Brazed Joint

**Figure 2:**
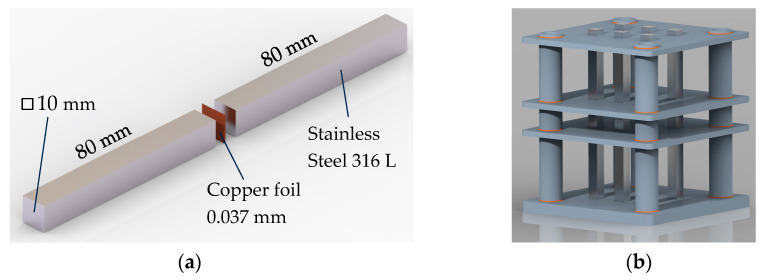
Specimen preparation: (**a**) Uniaxial tension specimen; (**b**) assembly for vacuum brazing.

In this study, stainless steel 316L was used as a plate material (see Table 1). Copper was used as a connecting material, which connects plates together inside the heat exchanger. When preparing the specimens for quasi-static and fatigue tests, two bars made of stainless steel were vacuum brazed using a copper foil with a thickness of 0.037 mm (see Figure 2) made of pure copper (99.99%). The complete procedure of vacuum braising consisted of six stages (vacuuming, heating, solidification, slow cooling, fast cooling and room pressure). At the copper solidification temperature (≈1150 °C), the austenitic stainless steel came into the recrystallisation zone. To mitigate the level of recrystallisation and, consequently, the mechanical properties changes, the time of the solidification phases must be shortened as much as possible, which depends on the wettability and capillarity between copper and austenitic stainless steel, the geometry of the brazing area, etc.

### 2.2. Experimental Testing of Brazed Joints

Experimental testing for both quasi-static and fatigue tests was performed on the Zwich/Roel Vibrophore 100 testing machine. The quasi-static tests were displacement-controlled using the mechanical extensometer with a constant displacement rate of 0.5 mm/min up to the final fracture of the specimen. Four quasi-static tensile tests were performed to obtain the engineering stress–strain diagram of the analysed brazed joint.

The fatigue tests were performed at a room temperature of 20 °C under a loading ratio *R* = 0.1 and a loading frequency of approximately 98 Hz. All tests were performed in the high cycle fatigue regime (HCF), which means that the maximum stress in each loading cycle did not exceed the yield stress of the analysed brazed joint.

### 2.3. Metallography

The samples were cut from tensile test specimens after fracture. The fracture surface was analysed in its original form without preparation. The fracture area of the tensile sample was prepared metallographically by mounting into a resin, grinding and polishing. After final polishing using a 3 µm diamond paste, the samples were analysed in the scanning electron microscope (SEM) Sirion 400 (FEI, Eindhoven, The Netherlands). In SEM, we also performed microchemical analysis using energy-dispersive spectroscopy—EDS (Oxford Analytical, Bicester, UK).

### 2.4. Computational Analyses of the Plate Heat Exchanger

**Figure 3:**
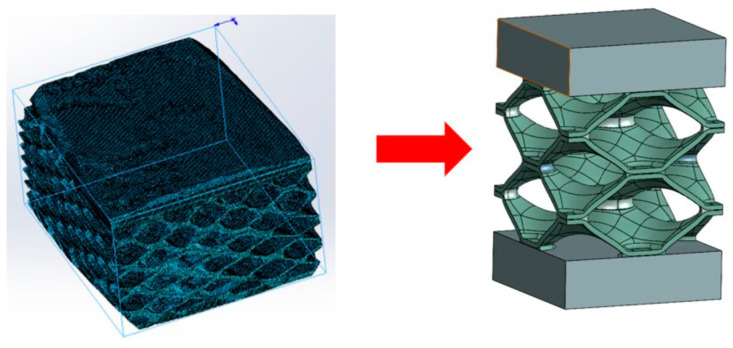
CT geometry (**left**) and simplified unit cell model (**right**).

The real geometry of the heat exchanger was captured by computed tomography (CT) technology. Here, the small part of real geometry was scanned using a ZEISS Xradia Versa 620 X-ray microscope (ZEISS Group, Jena, Germany) with an energy range of 30–160 keV X-ray source. The equipment had a resolution of 500 μm. Based on the cross-section slices from the acquired two-dimensional angular projections, the three-dimensional reconstruction was performed using the DragonFly Pro software (Version 2022.1). The scanned model was properly analysed in individual layers to find out where the non-brazed joints are located and what the surface and shape of the non-brazed joints are. Since we wanted to cover a larger area in our research, we decided to simplify and parameterise the numerical model. Thus, an analysis of the surfaces of brazed joints was made, where the average cross-sectional size of the brazed joint was determined. In the Ansys Space Claim (Version 2022 R2) and Solidworks software (Version 2022), a slightly more simplified geometry was created, which is shown in Figure 3.

**Figure 4:**
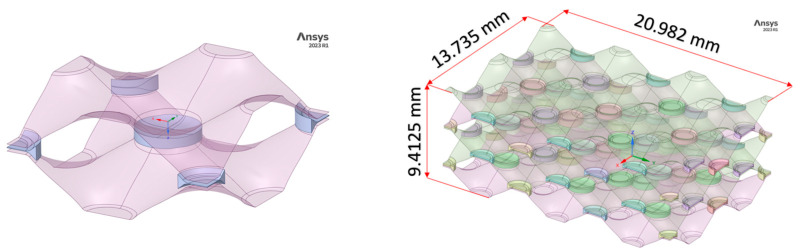
Unit cell of heat exchanger (**left**) and geometry of numerical parametric model (**right**).

As mentioned above, the aim of the research was to investigate the influence of non-brazed joints on the lifetime of the heat exchanger. With the desire to investigate as large an area as possible, a hybrid “solid-surface” model was created, where the brazed joints were dimensioned as solid models, and the geometry of the “honeycomb” was in the form of a surface model. Thus, a hybrid solid-surface geometry was created, which was then extended and parameterised. Figure 4 shows the unit cell and parametric model which was used for the subsequent numerical analysis.

**Figure 5:**
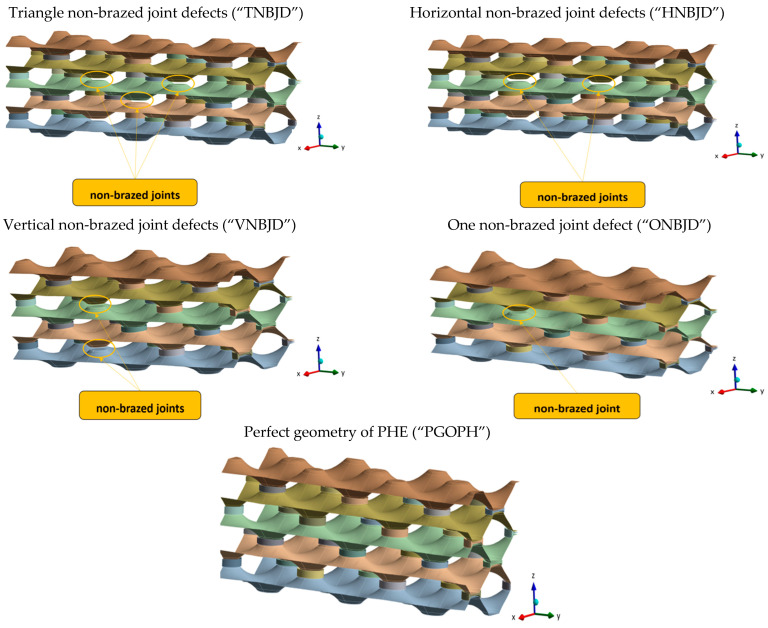
Parametric geometries of PHE.

For parametric numerical study, five different geometries were made. One of the geometries was modelled as a defect-free geometry, while the other geometries contained defects that were distributed in different ways within the numerical model. All geometries of parametric numerical models are shown in Figure 5, where each geometry is named with an abbreviation, which is a puzzle of the initial letters of individual words that define the geometry of each model.

**Figure 6:**
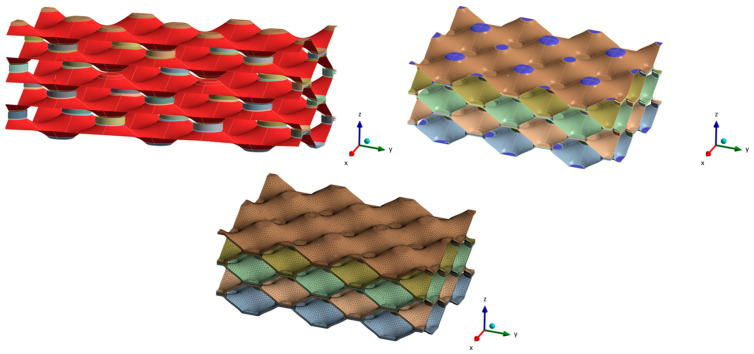
Prescribed boundary conditions and FE-mesh of the numerical model.

When creating the computational model, the material parameters obtained with experimental tests (see Section 2.2) have been considered. In the computational model, it was necessary to define contact points and, consequently, a “bonded contact”, which was created between the sheet metal and the brazed joints. Based on the theory of mechanical contact of two bodies, the brazed joint had the property of “contact body”, and the geometry of the sheet metal was defined as “target body”. The determination of the contacts was followed by the spatial discretisation (meshing) of the numerical model and the execution of a convergence analysis, based on which the global size of the finite element was determined. Thereafter, it was necessary to define the boundary conditions, which simulate the real loading conditions of PHE. The “fixed sup-ports” were prescribed on the lower and upper sides of the geometry of PHE (blue surfaces in Figure 6). The loading of PHE was prescribed in the form of a working pressure (15 bar). The pressure load was defined on the sheet metal and the locations where there were defects (i.e., non-brazed joints). At that point, we made another simplification because, in real conditions, the pressure load would affect the whole geometry, including the brazed joints. This simplification was used because the area of brazed joints is relatively small compared to sheet metal.

## 3. Results and Discussion

### 3.1. Quasi-Static Tensile Tests of Brazed Joint

**Figure 7:**
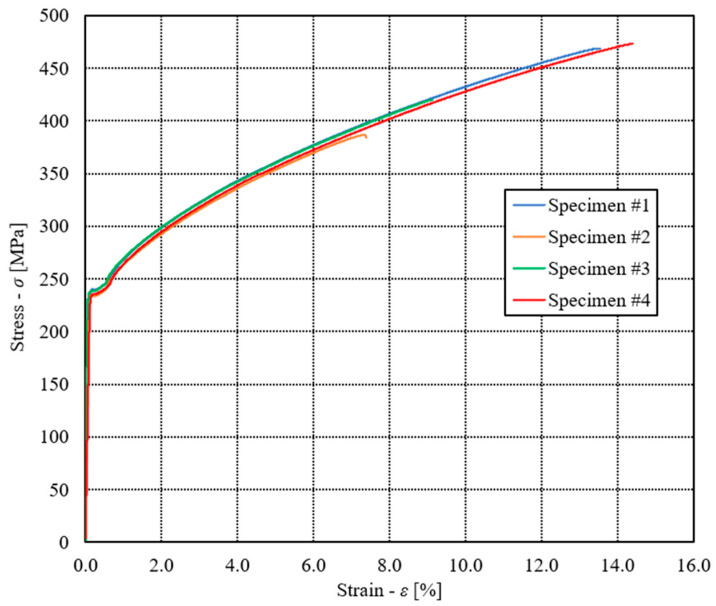
Engineering diagram σ-ε of quasi-static tests.

Figure 7 shows the engineering diagram σ − ε of quasi-static tests. Taking into account all the experimental results for all four tested specimens, it follows the following average values: Young’s modulus *E* = 198,387 MPa, Poisson’s ratio ν = 0.28, Yield stress *R*_e_ = 237 MPa, ultimate tensile stress *R*_m_ = 437 MPa and elongation at break EL = 11.1%. It is clear that due to the very thin layer of copper foil (0.037 mm), the specimen’s stiffness is defined by the mechanical properties of stainless steel 316L.

**Figure 8:**
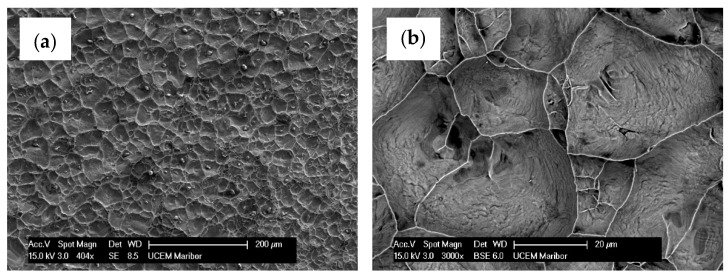
SEM images of the fracture surface of a tensile specimen: (**a**) lower magnification (secondary electron image); (**b**) higher magnification (backscattered electron image).

**Figure 9:**
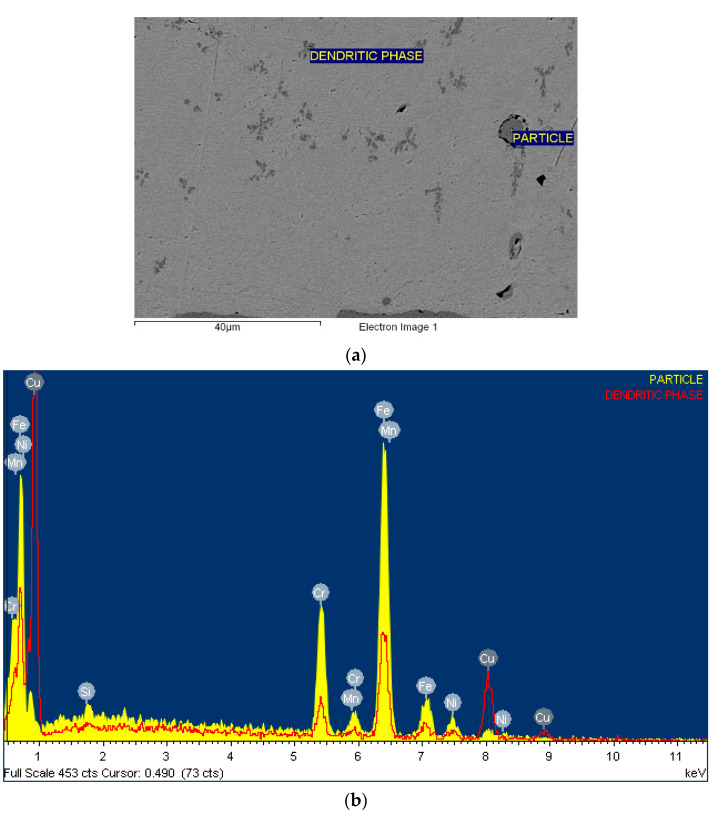
EDS-microchemical analysis result of the Cu foil: (**a**) SEM image of the analysed site, (**b**) Spectrums of the EDS-microchemical results of the particle in the Cu foil.

Figure 8 shows the SEM images of the fracture surface of a tensile specimen. The fracture has the characteristics of a ductile fracture. The dimples were evenly distributed over the entire fractured surface. It seems that they nucleated at intermetallic particles. Microchemical EDS analysis (see Figure 9) revealed the presence of particles in the remains of the broken Cu foil. In addition to the dendritic phase formed in the Cu matrix and containing the elements Cu, Fe, Cr, Mn, Si and Ni, irregularly shaped particles containing the elements Fe, Cr, Mn, Si and Ni and originating from the stainless steel substrate were also present.

**Figure 10:**
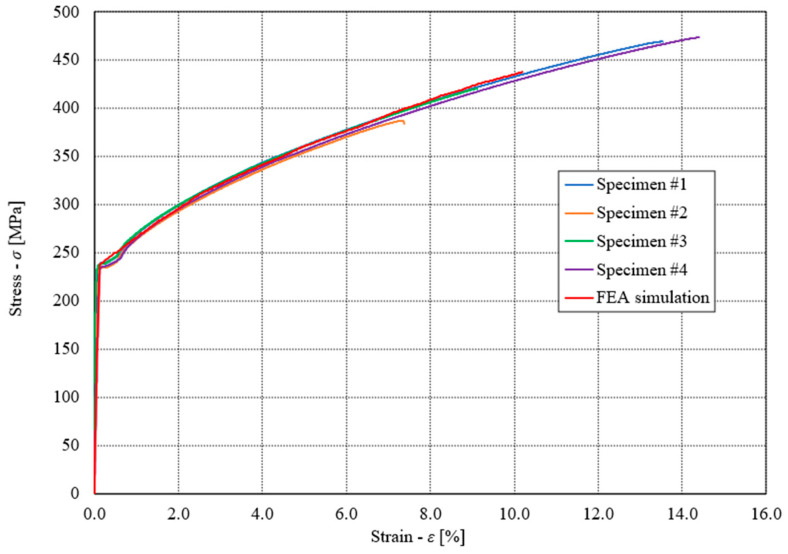
Experimentally and computationally obtained engineering diagram σ-ε.

The experimental results of quasi-static tensile tests (see Figure 7) were then considered to create the material model of the analysed brazed joint, which was used for the subsequent computational analyses of the plate heat exchanger (see Section 3.3). The numerical modelling of the analysed brazed joints was performed using the Ansys software, where the simplified geometry of the brazed joint specimen was applied for that purpose. In this case, the numerical model was built as a solid homogeneous piece of the material with the same cross-section area as already used in the experimental testing (see Figure 2). However, only the measuring length of the specimen (i.e., 50 mm) was considered for the subsequent numerical computations. Figure 10 shows the comparison between experimental and computational results for the engineering diagram σ − ε. It is evident that for all considered experimental tests, yield stress was around 237 MPa, which corresponds to the numerical simulations. In the elastic area (below yield stress), a very good match between experimental and numerical results was observed.

### 3.2. High Cycle Fatigue Tests of Brazed Joint

**Figure 11:**
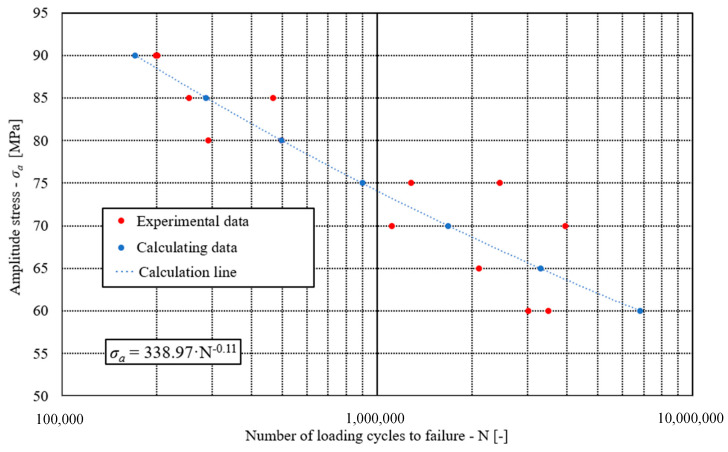
The S − N plot of the analysed brazed joints.

Fatigue tests have been performed on the same machine as quasi-static tests (Zwich/Roel Vibrophore-100, ZwickRoell, Ulm, Germany) using the same specimens (see Figure 2). The fatigue tests were performed at a room temperature of 20 °C under a loading ratio *R* = 0.1 and a loading frequency of approximately 98 Hz. All tests were performed in the high cycle fatigue regime (HCF), which means that the maximum stress in each loading cycle did not exceed the yield stress of the analysed brazed joint (i.e., *R*_e_ = 237 MPa). Following this limitation, the maximal tensile stress between (0.6 … 0.8)·*R*_e_ was selected. Figure 11 shows the S − N plot of fatigue tests where amplitude stress σ_a_ is given on the ordinate axis.

### 3.3. Computational Analyses of the Plate Heat Exchanger

As described in previous sections, the parametric simulations were performed to study how different configurations (different defects) affect the displacements, the equivalent von Mises stresses and the fatigue life of the analysed heat exchanger. Here, we were not focused on the absolute numerical values that we obtained in each numerical simulation but rather on how the numerical model responded to different load cases (different geometries) and how they differed from each other.

**Figure 12:**
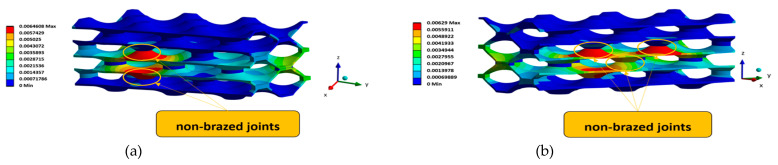
Displacements in the analysed PHE-structures in [mm]: (**a**) VNBJD structure; (**b**) TNBJD structure.

The displacements inside the heat exchanger depend mainly on the number of non-brazed joints, as shown by the comparative analysis of different numerical geometries. The results indicate that the absence of brazed joints in the vertical direction causes more movement and is a less favourable load case. Figure 12 shows the most critical configurations in regard to the displacement in the analysed PHE structures.

**Figure 13:**
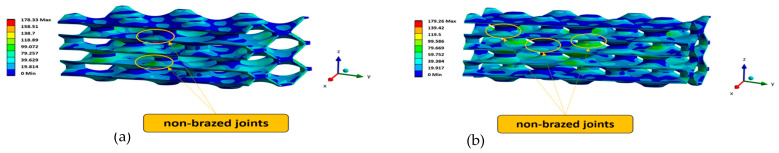
Von Mises equivalent stress in the analysed PHE-structures: (**a**) VNBJD structure; (**b**) TNBJD structure.

Figure 13 shows the von Mises equivalent stress in the analysed PHE structures for the two most critical configurations: VNBJD and TNBJD. In general, the parametric computational analyses have shown that the equivalent stress increases with the increase in the number of non-brazed joints, which confirms the thesis of load transfer to adjacent welded joints in the case of the presence of defects.

**Figure 14:**
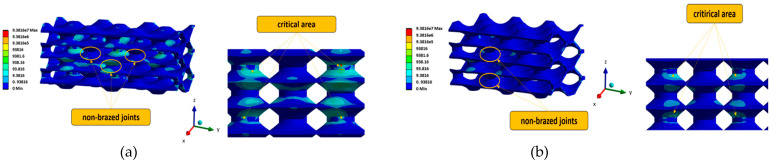
The fatigue life of two most critical geometries: (**a**) VNBJD structure; (**b**) TNBJD structure.

The determination of the fatigue life of a specific geometry was performed using the stress life approach. Considering this approach, it is assumed that failure occurs when the stress at a certain point reaches a critical value. Thus, based on the obtained computational results, a comparison with the strength properties of the material was made, and the fatigue life of the component was estimated. When determining the fatigue life of a component, it is also necessary to consider the mean stress effect. In the computational analyses presented in this study, the Goodman mean stress correction was applied considering the stress ratio *R* = 0.1. Furthermore, the fatigue strength reduction factor of 0.7 has also been considered in the subsequent computational simulations. The computational results (Table 2) confirmed the hypothesis that the expected fatigue life is dependent on the number and configuration of defects (i.e., non-brazed joints) inside the analysed PHE structure. Considering this assumption, it is evident from Table 2 that the longest fatigue life corresponds to the perfect geometry of PHE without defects (i.e., PGOPH). On the other hand, the shortest fatigue life belongs to the TNBJD structure, which represents the structure with three defects distributed in the form of a triangle. The graphical presentation of the obtained fatigue life of the two most critical geometries (VNBJD and TNBJD) is shown in Figure 14.

It is evident from Table 2 that the fatigue life of the analysed heat exchanger is dependent mostly on the geometry configuration of PHE considering the critical points (i.e., non-brazed joints), where the maximum equivalent stress appears in the vicinity of these points. However, it should be pointed out that the computational results were obtained considering the fatigue strength reduction factor of 0.7. As given in Ansys [33], this factor considers some additional influencing parameters (surface roughness, residual stresses, size effect, etc.), which may, in real structures, more or less differ if compared to the standardised test conditions. For comparison, Hayta et al. [32] used a value of 0.8, while Ma et al. [34] considered the value 0.3. Therefore, it is crucial to underscore that direct comparisons of computational results between different studies are questionable because of different assumptions regarding strength reduction factors and also due to the unique geometries inherent to each numerical model.

## 4. Conclusions

The computational analysis of brazed heat exchangers loaded with dynamic internal pressure is presented in this study. Based on the theoretical study, experimental testing, and comprehensive computational analyses, the following conclusions can be made:

- Considering the experimental results for all four tensile specimens, the material model of the analysed brazed joint has been developed;
- The fatigue tests of brazed tensile specimens were performed to obtain the S − N plot. The experimentally obtained S − N curve was then used for the subsequent computational analyses of PHE with different geometrical configurations (i.e., different defects inside the PHE);
- The computational results have shown that individual defects (i.e., non-brazed joints) significantly influence the displacement and stress distribution near these defects. Namely, each non-brazed joint increases the stress field around the surrounding brazed joints, which leads to a shorter fatigue life of the whole structure of PHE;
- The developed computational model can be further used for the fatigue analyses of different PHEs. However, the computational model for the subsequent numerical analyses should be improved, considering the effect of temperature and boundary conditions (i.e., the connection of the PHE to the other components) on the fatigue behaviour of PHE. Furthermore, the experimental testing of the whole structure of PHE should be performed to confirm the computational results.

## Figures and Tables

**Table 1:** The chemical composition of stainless steel 316L in wt. %.

C	Si	Mn	P	S	Cr	Mo	Ni	N
≤0.03	≤1.0	≤2.0	≤0.045	≤0.015	16.5–18.5	2.0–2.5	10.0–13.0	≤0.11

**Table 2:** Computational results for the fatigue life of analysed PHE structures.

Geometry Configuration	Fatigue Life *N* [Cycles]
PGOPH	42.7 × 10^6^
ONBJD	5.3 × 10^6^
HNBJD	2.0 × 10^6^
VNBJD	1.9 × 10^6^
TNBJD	1.7 × 10^6^

## Data Availability

The data presented in this study are available on request from the corresponding author.
